# Influence of rotator cuff preload on fracture configuration in proximal humerus fractures: a proof of concept for fracture simulation

**DOI:** 10.1007/s00402-022-04471-9

**Published:** 2022-05-17

**Authors:** Maximilian Lenz, Stephanie Kahmann, Mehdi Behbahani, Lenhard Pennig, Michael Hackl, Tim Leschinger, Lars Peter Müller, Kilian Wegmann

**Affiliations:** 1 Department for Orthopaedic and Trauma Surgery, Faculty of Medicine and University Hospital Cologne, University of Cologne, Joseph-Stelzmann Strasse 24, 50931 Cologne, Germany; 2 Department for Bioengineering, University of Applied Sciences Aachen, Heinrich-Mußmann-Straße 1, 52428 Jülich, Germany; 3 Institute for Diagnostic and Interventional Radiology, Faculty of Medicine and University Hospital Cologne, University of Cologne, Kerpener Strasse 62, 50937 Cologne, Germany; 4 Orthopaedic Surgery Munich, OCM Clinic, Steinerstrasse 6, 81369 Munich, Germany

**Keywords:** Proximal humerus fracture, Biomechanical simulation, Fracture configuration, Fracture simulation, Rotator cuff, Surgical training

## Abstract

**Introduction:**

In regard of surgical training, the reproducible simulation of life-like proximal humerus fractures in human cadaveric specimens is desirable. The aim of the present study was to develop a technique that allows simulation of realistic proximal humerus fractures and to analyse the influence of rotator cuff preload on the generated lesions in regards of fracture configuration.

**Materials and methods:**

Ten cadaveric specimens (6 left, 4 right) were fractured using a custom-made drop-test bench, in two groups. Five specimens were fractured without rotator cuff preload, while the other five were fractured with the tendons of the rotator cuff preloaded with 2 kg each. The humeral shaft and the shortened scapula were potted. The humerus was positioned at 90° of abduction and 10° of internal rotation to simulate a fall on the elevated arm. In two specimens of each group, the emergence of the fractures was documented with high-speed video imaging. Pre-fracture radiographs were taken to evaluate the deltoid-tuberosity index as a measure of bone density. Post-fracture X-rays and CT scans were performed to define the exact fracture configurations. Neer’s classification was used to analyse the fractures.

**Results:**

In all ten cadaveric specimens life-like proximal humerus fractures were achieved. Two III-part and three IV-part fractures resulted in each group. The preloading of the rotator cuff muscles had no further influence on the fracture configuration. High-speed videos of the fracture simulation revealed identical fracture mechanisms for both groups. We observed a two-step fracture mechanism, with initial impaction of the head segment against the glenoid followed by fracturing of the head and the tuberosities and then with further impaction of the shaft against the acromion, which lead to separation of the tuberosities.

**Conclusion:**

A high energetic axial impulse can reliably induce realistic proximal humerus fractures in cadaveric specimens. The preload of the rotator cuff muscles had no influence on initial fracture configuration. Therefore, fracture simulation in the proximal humerus is less elaborate. Using the presented technique, pre-fractured specimens are available for real-life surgical education.

**Level of Evidence:**

III.

## Introduction

Surgical treatment of proximal humerus fractures is technically challenging. Managing the fragments for reposition and fixation is critical. Especially complex fractures demand special skill-sets from the treating surgeon. Although unreconstructible osteoporotic fractures in the elderly usually undergo prosthetic replacement, still open reduction and internal fixation (ORIF) are a sensible alternative, not least due to relevant numbers of complications in prosthetic replacement of the proximal humerus [1–4]. Even in case of prosthetic replacement of the proximal humerus in fractures, fixation of the tuberosities is a demanding procedure. Moreover, complex fractures in younger patients due to high-impact trauma is seen. Hence, there is reasonable interest in surgical training courses that focus on operative techniques for osteosynthetic reconstruction of proximal humerus fractures. A viable improvement for such courses is the use of pre-fractured human cadaveric specimens [5, 6]. Therefore, a reliable technique to simulate fractures of the proximal humerus is desirable. The basic fracture planes occur between the humeral head, the greater tuberosity, the lesser tuberosity and the shaft itself. These typical fracture configurations are seen in complex III and IV-part fractures of the proximal humerus and are found in around 21% of patients [7]. Regarding fracture mechanism, Edelson et al. published the “parachute reflex”—a fall onto the elevated arm at 90° of abduction—as a common cause of fracture morphology and fragment configuration [7]. Hence, a useful simulation of these fracture mechanisms has to reproduce such a compression of the proximal humerus against the scapula. Moreover, as the rotator cuff tendons are attached to the greater tuberosity and to the lesser tuberosity, there might be an additional role of these structures regarding the generation of realistic fractures of the proximal humerus.

The aim of the presented cadaveric study was to simulate proximal humerus fractures and to analyse the influence of muscle pull via the rotator cuff on the generated lesions. We hypothesized, that rotator cuff pull does have an influence on fragment distribution and fracture configuration.

## Materials and methods

### Preparation and testing

For the biomechanical analysis, ten fresh-frozen human cadaveric specimens of the shoulder joint from ten body donors (6 left, 4 right; 6 female, 4 male; average age 73.6 years, range 49–95 years) were available. Before testing, the specimens had been inspected for an intact rotator cuff, cuff deficient specimens were not included for the study (3 were excluded). The specimens were stored at – 20 °C and thawed for at least 12 h before preparation. The humeral shaft had been cut at 20–30 cm distally to the humeral head, the clavicle was attached to the acromion via an intact ac-joint. The scapula was intact. The ten specimens were randomly assorted to two groups of five, in one group with preloaded rotator cuff tendons and another group without rotator cuff preloading.

**Fig. 1:**
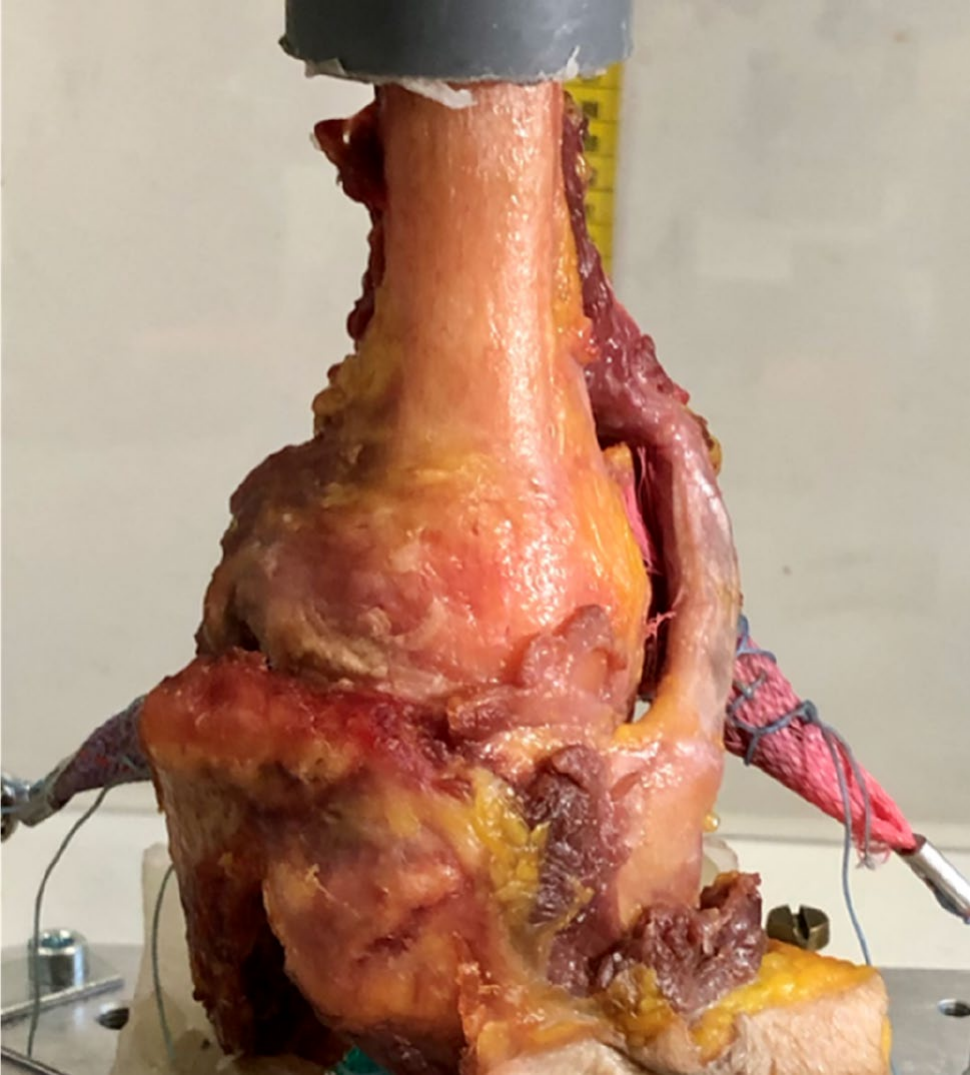
Schematics of the fracture simulation set up: the distal end of the humerus and the shortened scapula were potted in PMMA. The proximal humerus was positioned in 90° abduction and 10° of internal rotation with the glenoid positioned horizontally. Each rotator cuff tendon (SSP/ISP/SSC) was augmented with fingertraps and FiberWire

The distal humeral end was debrided from tissue and fixed in poly-methyl-methacrylate (PMMA) to be mounted into a custom-made fracture simulator. For the preparation of the rotator cuff tendons, complete detachment of the deltoid muscle was performed. The supraspinatus muscle (SSP), infraspinatus muscle (ISP) and subscapularis muscle (SSC) were prepared while the teres minor muscle (TEM) was not used for preloading. The origin of the muscles was proximally detached from the scapula and the distal tendinous part was isolated (supraspinatus fossa, infraspinatus fossa and subscapular fossa). Each tendon was sutured into a tubular plastic net with several stitches close to the insertion at the humeral footprint using FibreWire (Arthrex Fl. USA). In the preloaded group, each tendon was loaded with hanging 2 kg weights (19.8 N), a preload used in comparable studies [8–10] (Fig. 1).

The joint capsule was left intact, with the long head biceps tendon cut distal to the bicipital groove. In all specimens, the scapula was cut 4 cm laterally to its medial margin, allowing stable fixation of the scapula body, without using excessive amounts of epoxy resin into a rectangular bloc.

The prepared specimens were mounted into the custom-made drop-test bench, which was used in previous work [16, 17]. The test stand consisted of a steel frame using two central bars and a steel base plate (1.5 × 1.5 m). The height-adjustable cross beam had two gliding holes, thus, the cross beam fell onto an impact punch conducted by the two steel bars. The impact stamp, which was guided by two bars, had two endplates, one superior for the cross beam to hit onto and one inferior for mounting the specimen. The base plate of the potting cylinder was mounted on the inferior plate of the impact punch which transmitted the axial force directly onto the specimen. The opposite end of the specimen was fixed onto a steel table, which was fixed on the solid base plate using metal tracks. For fracture simulation in the present study, the scapula was fixed onto the baseplate of the fracture simulator, while the humeral shaft was mounted onto the potting cylinder in the upper segment of the simulator. The specimens were positioned in 90° abduction and 10° of internal rotation. Accordingly, the axial kinetic energy was calculated as $E$ = $\frac{1}{2}$ mv^2^ $(E$ = energy, $m$ = mass $v$ = velocity) with v calculated as $v=\sqrt{2gh}$ (g = 9,81 m/s, h = dropping height). The specimens were compressed with a mean of 142 Joule (range 127–156 J) as 17 kilos of weight were used as standard weight for dropping and drop height being determined by the remaining length of the humeral shaft. After fracture simulation, the specimens were immediately frozen at –20° Celsius without altering the fracture configuration for later radiographs and CT scans.

### Video documentation and imaging

High-speed resolution videos of the fracture simulation processes of two specimens in each group were acquired using a Fastcam SA1.1 High-Speed Video System Camera (Photron Inc., San Diego, CA, USA), creating videos with a frame rate of 3 and 30 frames per second (fps). X-ray was performed using an Exposcop 8000 Endo image intensifier (Ziehm Imaging, Nürnberg, Germany) with standard a.p. and lateral views. According to the technique by Spross et al. [11], deltoid-tuberosity measurements were performed on radiographs. Post-fracture CT scans were acquired using a commercially available 256-slice multi-detector CT scanner (iCT; Philips Healthcare, Best, The Netherlands). Sagittal and frontal planes were reconstructed using the manual Multiplanar Reconstruction (MPR) tool in the institutional picture archiving and communication system (IMPAX EE; Agfa Healthcare N.V., Mortsel, Belgium). 3D reconstructions were created using the vendor’s proprietary image viewer (CT viewer; Philips Healthcare, Best, The Netherlands). We analysed the fracture lines in a manner published by Hasan et al. [12]. We manually transcribed the fracture lines of the CT scans using the GNU Image Manipulation Program (Version 2.10.14, FSF, California, USA) with multiple layers matching the CT scans in size and position. All drawn fracture lines were than adapted and matched to an exemplary single layer of the CT scans.

### Statistical analysis

The donors’ specific data such as age, sex, deltoid-tuberosity index and technical measuring data were documented. Descriptive statistics were used to summarize the mean values and standard deviations (± SD). For non-parametric analysis (age, deltoid-tuberosity index, drop height, kinetic energy), Wilcoxon signed-rank test was performed to detect any statistically significant differences. The level of significance was set to *p* < 0.05. Classification of humerus fractures was performed by one orthopaedic surgeon (ML). In case of doubt, another orthopaedic surgeon was consulted (KW). The fracture configuration was classified according to the Neer Classification [13].

## Results

In this series, all specimens were fractured successfully using a custom-made fracture simulator. The fracture simulations resulted in III- and IV-part proximal humerus fractures. The specimens of both groups were similar regarding bone quality, showing minimal differences in the deltoid-tuberosity index (1.37 ± 0.085 vs. 1.38 ± 0.048; *p* = 0.76), age (82.2 ± 9.81 vs. 70.4 ± 15.2 years; *p* = 0.17) and sex (1 m:4f vs. 3 m:2f) in the preloaded group vs. the unloaded group, respectively (Table 1). The average drop height which is depending on the initial length of the remaining humerus shaft was 0.803 ± 0.035 m vs. 0.893 ± 0.053 m, respectively (Table 1). Therefore, specimens were hit by a mean of 142 Joule of kinetic energy (149 vs. 134 Joule). There were no statistically significant differences between the two groups in aspect of age (*p* = 0.131), kinetic energy (*p* = 0.08) or deltoid-tuberosity index (*p* = 0.68).

**Table 1:** Standard characteristics of the preloaded group (above) and the unloaded group (below) with the numbered specimens, the mean values and standard deviations (SD) are presented

Specimens’ characteristics							
	Side	Age (years)	Sex	Drop height (m)	Kinetic energy (J)	Deltoid-tuberosity index	Fracture parts
Pre-loaded group							
1	R	78	F	76	127	1.311	IV
2	L	95	M	78	131	1.486	III
3	L	90	F	80	135	1.272	III
4	L	72	F	83.1	139	1.394	IV
5	L	76	F	84.4	141	1.408	IV
Mean		82.2		80.3	134	1.374	
SD		± 9.81		± 0.035	± 5.81	± 0.085	
Unloaded group							
11	L	79	M	0.93	155	1.452	III
12	R	64	M	0.93	154	1.364	IV
13	R	89	F	0.89	148	1.330	III
14	R	71	M	0.94	156	1.422	IV
15	L	49	F	0.81	135	1.376	IV
Mean		70.4		0.9	149	1.388	
SD		± 15.2		± 0.053	± 8.87	± 0.048	

*L* Left, *R* Right, *M* Male, *F* Female

The fractures were classified according to the Neer Classification using CT scans. Within the preloaded group, two III-part fractures and three IV-part fractures were found. The unloaded group had the same results as two III-part fractures and three IV-part fractures were found.

**Fig. 2:**
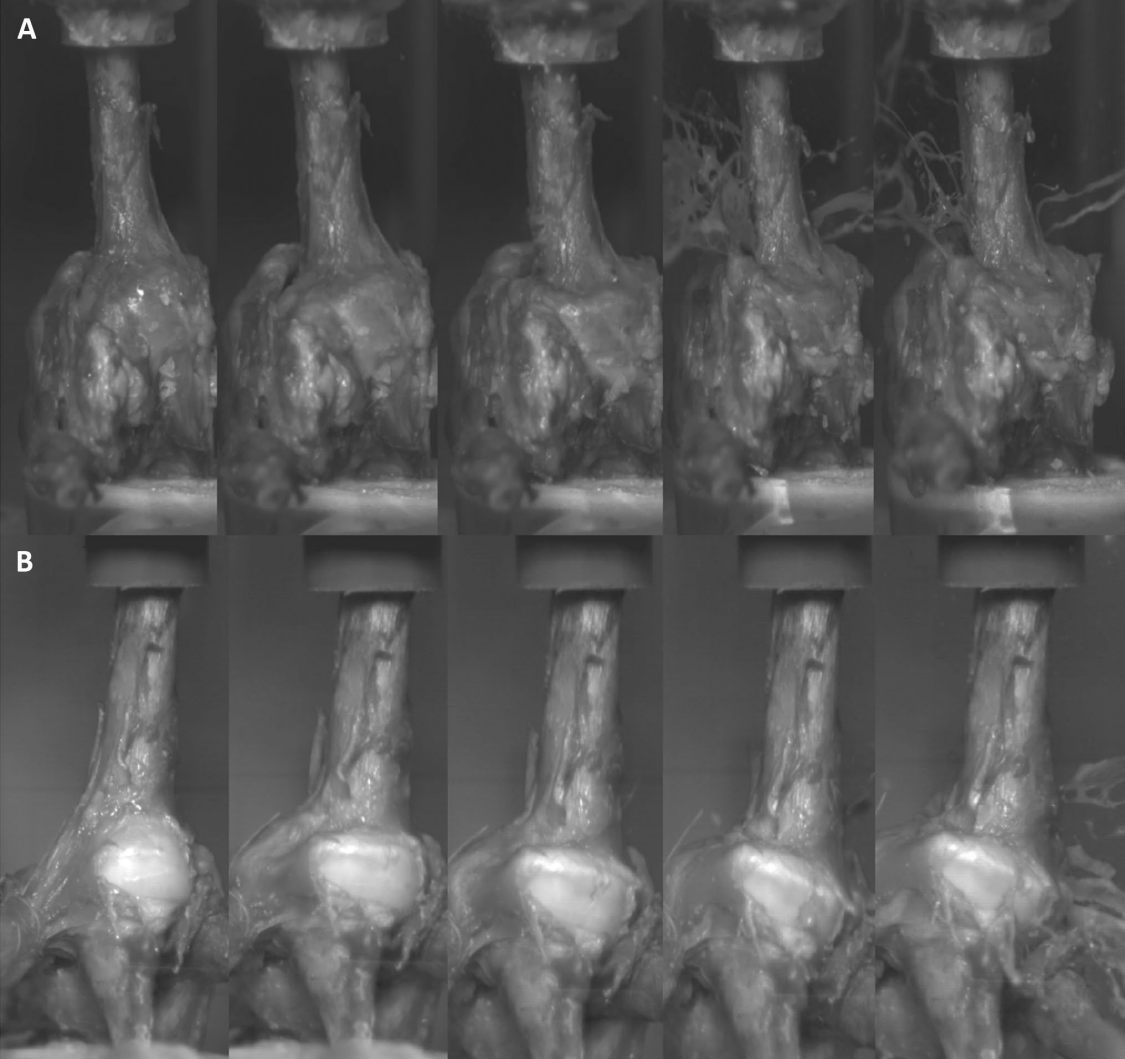
High-speed images of **A** unloaded specimen and **B** specimen with preloaded rotator cuff tendons. Typical shield fracture aspect seen in the head fragment of **A** and **B**

When acquiring the high-speed imaging we could find typical recurring steps in the fracturing process: a first impact was observed when the axial force hit the humeral shaft which struck the humeral head resulting in the first fracture line almost circular around the head (Fig. 2).

**Fig. 3:**
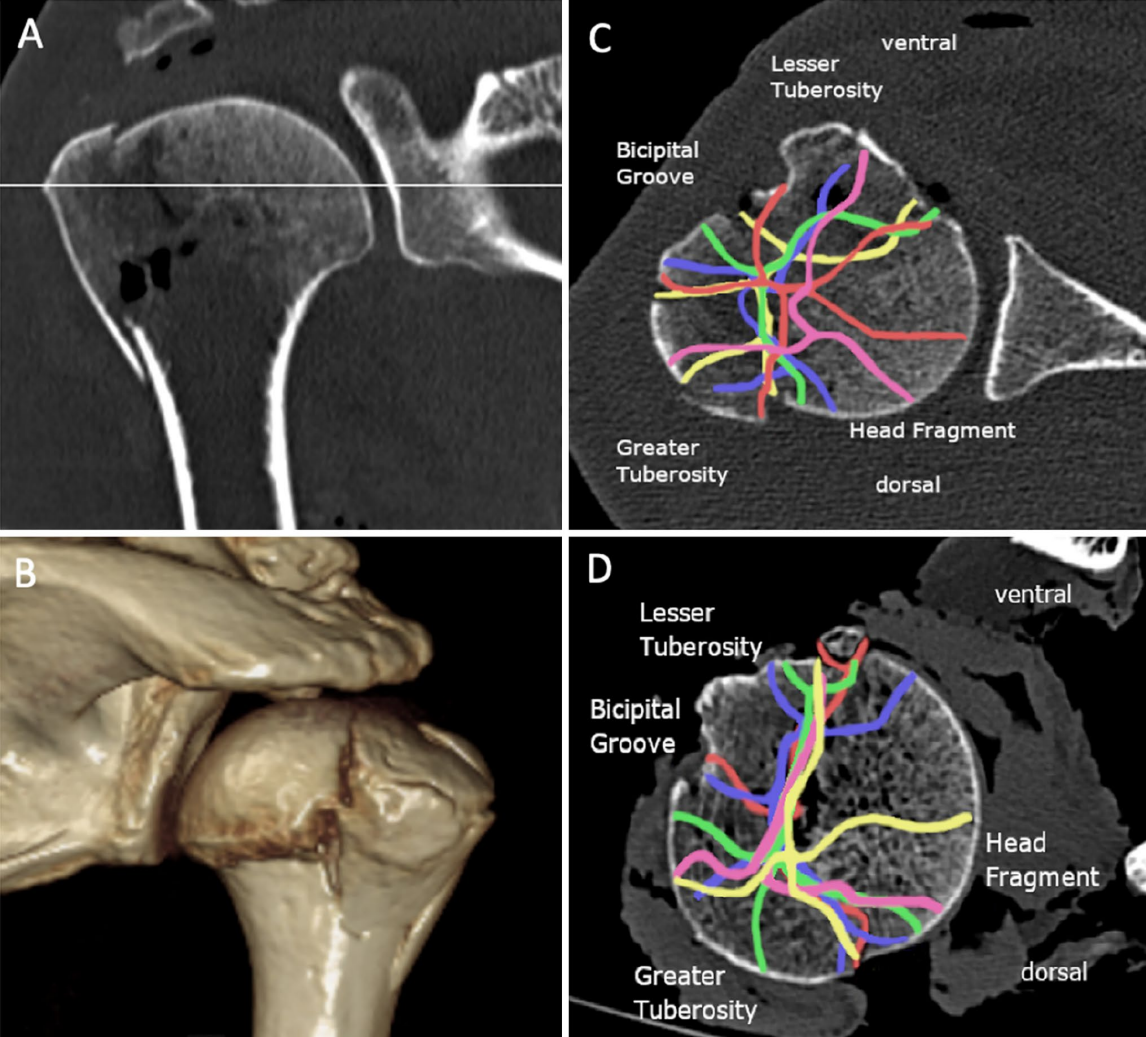
Analysis of fracture line configuration: **A** shows the standardized layer selection at the height of the greater tuberosity. **B** shows three-dimensional verification of fracture configuration. **C** marks the unloaded group and fracture lines, with the color differing from each specimen (soft tissue covering fully the glenohumeral joint) and **D** shows the preloaded group and the fracture line configuration with the color differing from each specimen (soft tissue partially removed)

The continuing force compressed the humeral head against the glenoid. When the humeral head was impacted into the glenoid convexity, both, the greater tuberosity and the lesser tuberosity abut onto the glenoid’s rim. Hence, the hard glenoid bone acted as anvil shearing off the greater and lesser tuberosity from the central humeral head part creating a longitudinal shear which resulted in a vertical fracture line along the shaft (Fig. 2). While the head fragment is set in the glenoid convexity, the greater and lesser tuberosities abut the acromion and are separated further. With the continuing impaction of the humerus shaft creates either varus or valgus deformity. The described process of fracture formation was observed in both groups during the high-speed imaging. Moreover, the typical configuration of fracture lines was also found in the CT scans (Fig. 3).

Analysing the fracture lines as described in the methods section, we found a typical distribution of fracture lines mainly between head and greater tuberosity in almost all proximal humerus fractures. The fracture lines closest to the intertubercular (bicipital) groove were mainly located in the greater tuberosity fragment omitting the groove itself.

## Discussion

The present study shows the creation of proximal humerus fractures by inducing a high energetic impulse onto human cadaveric specimens. By the use of a custom-made fracture simulator, we were able to simulate reproducible proximal humerus fractures by compression of the proximal humerus against the fixed scapula. The injury mechanism itself, the cortical bone thickness and bone mineral density as well as the given bony anatomy may contribute to the fracture configuration. Moreover, as the exact patho-mechanism of fracture configuration in proximal humerus fractures has not yet been fully understood, we analysed the influence of the rotator cuff preload. In the given in-vitro setting, the rotator cuff pull had no influence on the fracture configuration in proximal humerus fractures.

Simulation of proximal humerus fractures requires the imitation of the typical indirect injury mechanism which usually is a fall onto the 90° abducted arm in a lateral or ventral direction. This mechanism is also stated as “parachute reflex” and typically found in the stumbling fall in the elderly which leads to complex III or IV-part fractures with either varus or valgus impaction [1, 12]. We were able to generate life-like fracture configurations in proximal humerus by simulation of axial impact against the fixed scapula.

In the given in-vitro setting, the fracture lines followed a certain pattern: one fracture line is seen between head fragment and greater tuberosity, predominantly at the border of joint capsule. Another fracture line in IV-part fractures divides the greater and the lesser tuberosity which is described by Edelman et al. as “shield fracture” as worsening and progression of a III-part fracture. The fracture line omits the bicipital groove with the majority of the fracture lines passing through the fragment of the greater tuberosity which is conclusive to previous studies [7, 12]. The bicipital groove shows greater cortical bone thickness compared to the tuberosities [14]. Hepp et al. showed also lowest bone mineral density (BMD) in the central portion and in both tuberosities [15]. Furthermore, Tingart et al. demonstrated that BMD was 30% less in the greater tuberosity compared to the lesser tuberosity [16]. Therefore, fracture lines omitting the bicipital groove and passing through the side of the greater tuberosity may be largely determined by the BMD and cortical thickness. Overall mean values of the deltoid-tuberosity index measured by the technique by Spross et al. were 1.38 (tendons) vs 1.37 (control). This index shows correlation and predicts actual bone mineral density and cortical thickness with < 1.4 as cut off for minor bone quality. According to the recent literature and our findings, fragment distribution in proximal humerus fracture, is a result of the bony anatomy and the bone density, rather than due to muscle pull [17].

The typical fracture line between greater tuberosity and head fragment may be caused by the glenoid rim or the cortical thickness at the articular margin which was found to be thinnest in cadaveric specimens [14]. Additionally, the glenoid bone acting as an anvil and dissipating forces through weaker areas of thin cortical bone. For both groups, two head split fractures were seen. The fracture lines of the head fragments showed highest heterogeneity compared to the other fragments (Fig. 3). Head-split fractures are probably caused by further impaction of the shaft splitting the head fragment when centrally hitting into the glenoid convexity (Fig. 3). If the head fragment is only partially covered by the glenoid convexity, the head is split at the edges of glenoid cover. Based upon these anatomical and radiological results, our study showed highly similar results in all aspects of fracture configuration of the proximal humerus in both groups (Figs. 2 and 3).

In another cadaveric study, fracture configuration showed similar results to our study with only the proximal humerus tested [17]. Majed et al. found correlation of fracture’s configuration to cortical thickness and the “parachute reflex” as mechanism [17]. Their high-speed imaging of fracturing showed similar fracture lines and fragment configuration compared to our results. They recognized the “parachute reflex” to produce shield and head-spilt fractures [14]. The conclusion that fracture morphology can be determined by bony anatomy is, therefore, supported, especially as their specimens had no soft tissue. Analysis of isolated fractures of the greater tuberosity support the influence of the bony anatomy in proximal humerus fractures [18]. Treatment of proximal humerus fractures in the elderly requires knowledge and experience in reduction and fixation techniques as well as implantation of a glenohumeral prothesis. Therefore, surgical training using pre-fractured specimens to teach and learn surgical approaches, operational reduction and fixation techniques or implantation of a prothesis, provides the most realistic surgical experience. Although the creation of proximal humerus fractures of specimens is technically demanding and the specimens as well as the test-bench are associated with high costs, the candidates benefit from the life-like teaching situation. As the rotator cuff preload showed no effect on the fracture configuration, this simplifies the creation of proximal humerus fractures for cadaveric surgical training. Moreover, the specimens do not need further soft tissue preparation and candidates can train on specimens with intact skin envelope. This results in a more realistic surgical training as the different surgical approaches can be trained on top of the reduction techniques.

Several limitations must be mentioned. The sample size is small due to high costs related to purchase and preparation of cadaveric specimens, performing CT scans and high-speed camera imaging. In-vitro setting of fracture analysis can only imitate reality as accurate as possible. Due to notable limited space for placing the weights, strength vectors of the tendons varied slightly from the natural course, whilst best compromise to actual anatomical vectors was applied. Furthermore, the joint in our simulation is fixed in a rigid construction while in reality, the joint itself with scapula, clavicle and humerus being in a semi-rigid situation allowing compensatory movements. Moreover, our results of proximal humerus fractures are not transferable to younger patients’ fractures without limitation. Regarding bone quality measurement, the prepared joints showed significant damage to the extent that there was insufficient continuous bone and aerial artefacts pre-empting CT analysis using Hounsfield units (HU) in the humeral head.

In conclusion, a high energetic axial impulse can induce life-like proximal humerus fractures in cadaveric specimens. By applying biomechanical fracture simulation using a custom-made drop-test bench, we provide the first data available investigating the influence of the rotator cuff preload on fracture configuration. In the given in-vitro setting, the preloaded rotator cuff had no altering influence on fracture configuration. Therefore, fracturing is thought to be determined primarily by joint anatomy and bone quality rather than muscle strength. The creation of fractures without further soft-tissue preparation facilitates fracture simulation and allows for an even better technical training for real-life surgical education.
